# Effects of reinforcing materials on durability of bone cement: in vitro experimental study

**DOI:** 10.1186/s13018-018-0799-9

**Published:** 2018-04-19

**Authors:** O. Karakus, O. Karaman, B. Gurer, B. Saygi

**Affiliations:** 1 Omer Halis Demir University Hospital, Nigde, Turkey; 2 Fatih Sultan Mehmet Teaching and Research Hospital, Petrolis distreet, rahmanlar st. Sumerevler bloc no: 26 Kartal, Istanbul, Turkey; 3 Omer Halis Demir University Hospital, Nigde, Turkey; 4 Maltepe University, Istanbul, Turkey

**Keywords:** Bone cement, Silica fumes, PMMA, Compression pressure measurement

## Abstract

**Background:**

Bone cement is one of the most commonly used products in orthopedic surgery. Among common indications for its use are total joint replacement, bone and joint reconstructions, fracture fixation, treatment of bone infections, and treatment of osteoporotic vertebral fractures. Endurance is still questionable. The aim of our study is to find out the effect of structure strengtheners on compression pressure measurements of bone cement.

**Methods:**

There were four groups in this study: group 1, 40 cm^3^ pure bone cement (PMMA); group 2, 40 cm^3^ bone cement with %25 titanium dust; group 3, 40 cm^3^ bone cement with %25 steel dust; and group 4, 40 cm^3^ bone cement with %25 silica fume mixtures were prepared. These mixtures were frozen in 6-mm-width, 12-mm-height molds in cylindrical shape. Axial compression was made to these molds.

**Results:**

Compression pressure measurements of the pure cement group ranged between 79.2 and 81.1 MPa; average was 80.25 ± 0.42 MPa. Measurements of titanium-added group ranged from 79.5 to 81.2 MPa; average was 80.46 ± 0.68 MPa. Steel-added group ranged from 79 to 82.2 MPa; average was 80.73 ± 0.57 MPa. For silica fume, measurements ranged from 89.1 to 91.4 MPa and average was 90.41 ± 0.57 MPa. The highest compression pressure values were gathered from the silica fume (*p* = 0.001).

**Conclusion:**

The construction reinforcer silica fume could be mixed with PMMA homogeneously and was superior to the other biocompatible materials that we had used in compression pressure tests. Beyond dispute, silica fume is a reinforcer which also increases the strength of the bone cement.

## 1.Background

Bone cement is one of the most commonly used biomaterials in orthopedics. This material, also known as polymethylmethacrylate (PMMA), has been used for more than 60 years in the field of orthopedics [1, 2]. Initially, bone cement was used in dentistry, then it was introduced by Charnley to hip replacement surgery by 1960s [3, 4].

The primary property that distinguishes biomaterials from other materials, used in engineering fields, is biocompatibility, which is the ability to function with the proper host response.

In the orthopedic surgery, stainless steel, cobalt-chromium alloys, titanium, and titanium alloys as metallic biomaterials; alumina, zirconia, and some porous ceramics as ceramic biomaterials; silicone, polyethylene, polyurethane, polypropylene, and polymethylmethacrylate as polymer biomaterials are widely used. In addition, carbon fibers are widely used to strengthen polymers [5–7].

On the other hand, in building constructions, reinforcing materials have been used in concrete to increase the load carrying capacity. The materials are preferred in orthopedic surgery because of their mechanical strength. Therefore, reinforcing materials might be added to bone cement in order to strengthen it. Common uses of polymethylmethacrylate (PMMA), bone cement, include total joint prosthesis, joint and bone reconstructions, bone infections, and treatment of vertebral fractures due to osteoporosis.

The hypothesis of present study is building reinforcing elements will also increase resistance of bone cement mechanically. The purpose of the study is to investigate the effects of different strengthening agents on bone cement durability.

## 2.Methods

Plastic (polyethylene terephthalate) tubular molds with a height of 12 mm and 6 mm in diameter were prepared as stated in the F451-99a code numbered Acrylic Bone Cement Standard Specifications section [8] of the American Society for Testing and Materials (ASTM). The molds had two covers. For the removal of the prepared PMMA, a steel rod with a smooth surface of the same diameter as the mold was used. All samples were prepared at room temperature.

There were four groups in the study: group 1, 40 cm^3^ of pure bone cement (40 g of Surgical Simplex P bone cement); group 2, 40 cm^3^ of bone cement with 25% titanium powder added (Titanium 6Al-4 V Grade 3); group 3, 40 cm^3^ of bone cement with 25% steel powder (X2CrNiMo1812 -316 L); and group 4, 40 cm^3^ of bone cement with 25% silica fume (Sika MonoTop 610) mixtures were used.

Steel powder and titanium powder were obtained from TST (Medical Devices Industry and Trade Limited Company) branded plates.

**Fig. 1:**
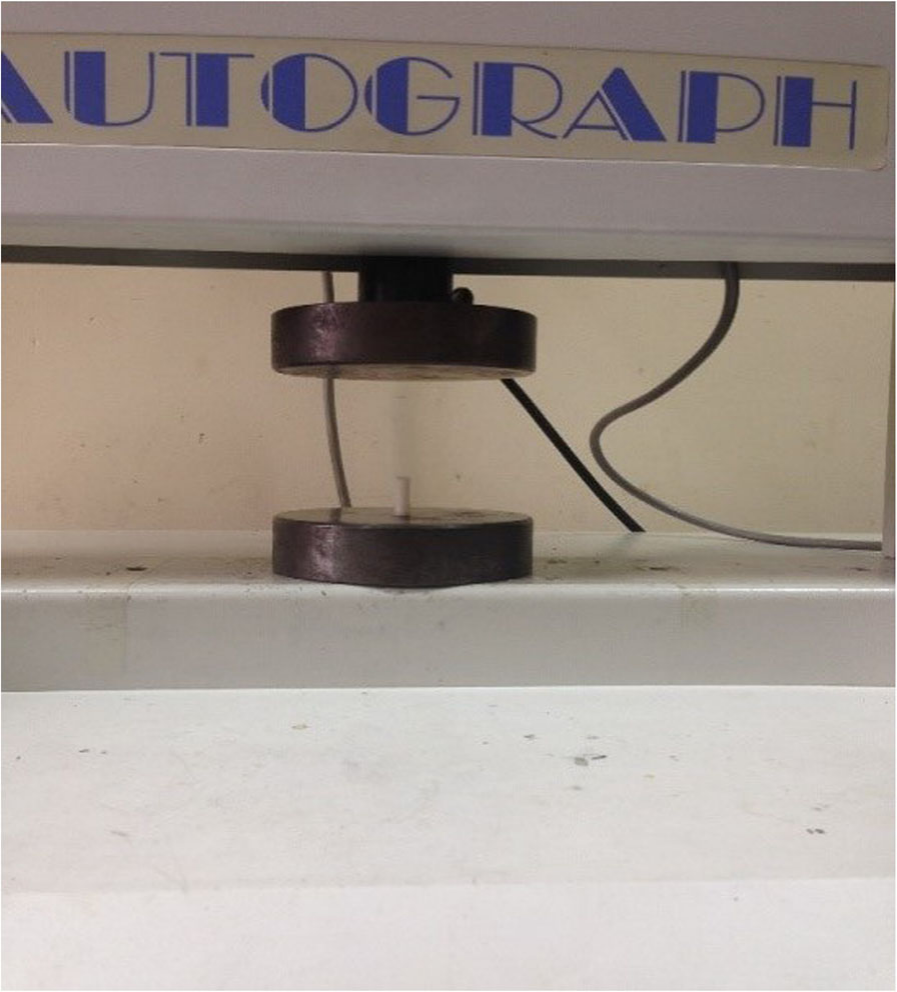
Frozen cement molds

A total of 40 g cement powder was mixed with 25% ratio 10 g silica fume, 10 g steel powder, and 10 g titanium powder in separate containers, mixing for 1 min to obtain different groups. Following the addition of liquid monomers, the mixture was mixed for a further 30 s, and kept for 90 s at room temperature. They were then filled into molds with manual pressure (Fig. 1). The lids were closed and the cement mold compressed with power grip. The power grip was loosened after 15 min, and lids were opened. With the help of a smooth surface steel rod with a diameter of approximately 5.5 mm, the cylindrical samples were removed from the cement mold. Their surfaces were smoothed with number 0 sand papers. Four different groups consisting of 25 samples in each group were obtained. The cylindrical samples prepared as 100 pieces for all groups were reviewed macroscopically. Samples with 10% or higher level of cracks and gaps on the surface were excluded from the study [8, 9].

The samples were stored in closed containers at 20 ± 2 °C room temperature for 4 weeks. For the compression tests carried out at the end of the 4-week process, the method stated in the F451-99a code numbered Acrylic Bone Cement Standard Specifications section of the American Society for Testing and Materials (ASTM) was used. Axial compression tests were carried out with 10,000 N capacity press-tension device (SHIMADZU 10KN AGS-J; made in Kyoto, Japan). The pressing speed was set to 5 mm/min, and the samples were pressed until breaking point [9].

NCSS (Number Cruncher Statistical System) 2007 (Kaysville, UT, USA) was used for statistical analysis. When evaluating the study data, in addition to descriptive statistical methods (mean, standard deviation, median, minimum, maximum), in the comparison of three or more groups not showing normal distribution, Kruskal-Wallis test, and in the determination of the group leading to the difference the Mann-Whitney *U* test was used. Significance was considered at levels *p* < 0.01 and *p* < 0.05.

## 3.Results

The study was conducted with a total of 100 samples. The compression pressure measurements of the pure cement group differed between 79.2 and 81.1 Mpa, with an average of 80.25 ± 0.42 Mpa. For those with titanium added, these values differed between 79.6 and 81.2 Mpa, with an average of 80.46 ± 0.68 Mpa. For steel, these values differed between 79 and 82.2 Mpa, with an average of 80.73 ± 0.57 Mpa. For silica fumes, these values differed between 89.1 and 91.4 Mpa, with an average of 90.41 ± 0.57 Mpa. The lowest compression pressure was measured with pure cement group. The highest compression pressure was measured with silica fumes (*p* = 0.001) (Table 1).

**Table 1:** Compression pressure measurement values according to groups

	Pressure values (MPa)		*p*
	Min-Max (median)	Mean ± SD	
Group 1 (*n* = 25)	79.2–81.1 (80.2)	80.25 ± 0.42	*0.001*
Group 2 (*n* = 25)	79.5–81.2 (80.7)	80.46 ± 0.68	
Group 3 (*n* = 25)	79.0–82.2 (80.5)	80.73 ± 0.57	
Group 4 (*n* = 25)	89.1–91.4 (90.5)	90.41 ± 0.57	

Kruskal-Wallis *H* test (*p* < 0.01)

A statistically significant difference was determined between the compression tests carried out on pure cement and those using titanium; resistance to pressure were shown to be increased with the usage of titanium (*p* = 0.007; *p* < 0.01).

A statistically significant difference was determined between the compression tests carried out on pure cement and those using steel, with the pressure shown to have increased with the usage of steel (*p* = 0.030; *p* < 0.05).

A statistically significant difference was determined between the compression tests carried out on pure cement and those using silica fumes, with the pressure shown to have increased with the usage of silica fumes (*p* = 0.001; *p* < 0.01).

There was no statistically significant difference between the titanium group and steel group compression pressures. (*p* = 0.240; *p* < 0.05).

A statistically significant difference was determined between the compression tests carried out on titanium and silica fumes, the pressure was shown to have substantially increased with the usage of silica fumes (*p* = 0.001; *p* < 0.01).

A statistically significant difference was found between the compression tests carried out on steel and those using silica fumes, the pressure increased substantially in the favor of silica fumes (*p* = 0.001; *p* < 0.01) (Table 2).

**Table 2:** Paired comparison of groups

	*p*
Cement Gr–Titanium Gr	0.007
Cement Gr–Steel Gr	0.003
Cement Gr–Silis Fume Gr	0.001
Titanium Gr–Steel Gr	0.240
Titanium Gr–Silis Fume Gr	0.001
Steel Gr–Silis Fume Gr	0.001

## 4.Discussion

Many studies have been conducted to carry out research on the durability of PMMA in the past. The impact on mechanical strength of the addition of antibiotics to bone cement has been investigated in many studies [10–12].

The material obtained by rapidly cooling to condensate the gas occurring during the production of silica metal or silicon metal alloys, containing 85 to 98% silica containing very fine particles with an amorphous structure is called “condensed silica fume” or “silica fume.” This material is also referred to as “microsilica” or “silica powder” or “silica fume.” Silica fumes, due to its amorphous structure and being a very fine grained material, containing high amounts of SiO2, is an excellent pozzolanic material [13].

The maximum antibiotic concentration can be added to bone cement without impacting mechanical strength, and the negative impact on mechanical strength of high antibiotic doses mixed have been shown in many studies. In a study carried out by Gögüs et al., the highest safe dose of teicoplanin to be added to 40 g Surgical Simplex P bone cement in third-generation cement application and preparation conditions was shown to be 1600 mg. When exceeding these doses, a significant decrease in cement durability is shown [14].

In contrast to the common studies carried out with the addition of antibiotics to PMMA, we wished to calculate the mechanical strength change when reinforcing materials, widely used in the construction industry, had been added to bone cement. The reason for this was the desire to focus on the possibility of mitigating the negative impact of additives such as antibiotics added to the cement for therapeutic purposes with reinforcing elements. Therefore, silica fumes and steel, which are used as strengtheners in the construction industry, along with titanium used in modern orthopedics due to its high level of biocompatibility had been chosen for evaluation in the current study.

The steel, which is used in both the construction industry as well as orthopedic surgery, and titanium are structurally similar. However, both these materials do not always give excellent results in terms of durability in the orthopedic surgery industry, leading to implant failure from time to time. In studies of sectors outside of the orthopedic industry, high durability had been obtained with the addition of silica fumes to cement. Therefore, we included silica fumes in order to see its effect on bone cement; however, its biocompatibility is also still unknown.

**Fig. 2:**
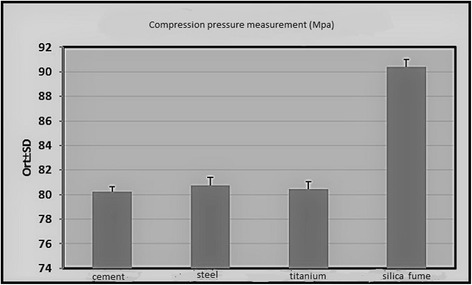
Compression pressure measured values according to groups

When all the groups in the study were examined, the average compression pressure values of pure bone cement had been found to be 80.25 Mpa. With the addition of titanium, an average value of 80.46 MPa, with the addition of steel 80.73 MPa, and the addition of silica fumes 90.41 MPa had been achieved. The highest average MPa value in our study was determined to be with the addition of silica fumes to bone cement. This was followed by steel, titanium, and pure bone cement, respectively. In the compression pressure tests carried out, the cement containing silica fumes was found to be statistically significantly superior to pure cement, cement containing steel, and cement containing titanium (Fig. 2).

The durability of stainless steel was higher in comparison with titanium, in the elastic modulus curve. However, in the compression pressure analysis tests obtained with the addition to PMMA, no statistically significant difference could be found between the two materials.

About the characteristics of silica fumes, a study conducted on concrete compressive strength had been shown that silica fumes had a high level of impact on compressive strength. Concrete containing silica fume had been shown to gain from 20 to 50% more strength when compared to control concrete [15]. When considering in terms of mechanical strength, the puzzolanic effect of silica fumes were said to be important in strengthening the aggregate-cement paste contact surface, known as the weakest link in cement [16]. It is known that silica fumes fill the micro gaps in concrete, providing resistance to many outside factors which negatively impact durability. By reducing permeability tenfold, resistance against many chemical and biological effects, which are dangerous in terms of concrete such as carbonation and alkali-sulfate reaction, was increased [17]. Silica fumes can also be used in the orthopedic surgery industry according to these properties.

The present study aimed to focus on orthopedic surgery cases which require stronger bone cement. The reinforcing materials can make new profit and improvement in filling of bone defects in arthroplasty revision cases, in need of stronger fixation of prosthesis in osteoporotic-ostenecrotic-rheumatic cases, and particularly in infected nonunions where rods prepared from antibiotic-loaded bone cement (ALBC) for a temporary period which could be shifted to permanent treatment if the rods might be prepared from reinforced antibiotic-loaded bone cement.

The current study is an experimental in vitro study. Weaknesses of it are that reinforcer’s biocompatibility, mixture ratio, and adherence capacity to bone and prosthesis were not evaluated.

## 5.Conclusion

In the present experiment, silica fume improved durability of bone cement superiorly in the compression tests and mixed homogeneously with PMMA compared to other biocompatible materials used as strengtheners. The biocompatability of silica fume and these kinds of reinforcers should be searched in order to view availability in the orthopedic surgery industry.

Resistance to compression of ALBC is shown to be lowered above certain concentrations of antibiotics (Ref). Reinforcing elements can overcome this durability loss and provide inclusion of more antibiotics into bone cement. Accordingly, this will provide new opportunities of more antibiotic concentrated and stronger ALBC spacers-rods-nails especially in the treatment of infected arthroplasty, infected nonunion, and osteomyelitis cases. As a result, this is a possible way to obtain mechanically strong and high-antibiotic release mixtures.

## Abbreviations

ALBC: Antibiotic-loaded bone cement
ASTM: American Society for Testing and Materials
NCSS: Number Cruncher Statistical System
PMMA: Polymethylmethacrylate
TST: Medical Devices Industry and Trade Limited Company
